# Regulated Expression of *miR-155* is Required for iNKT Cell Development

**DOI:** 10.3389/fimmu.2015.00140

**Published:** 2015-03-30

**Authors:** Alessia Burocchi, Paola Pittoni, Esmerina Tili, Alice Rigoni, Stefan Costinean, Carlo Maria Croce, Mario Paolo Colombo

**Affiliations:** ^1^Molecular Immunology Unit, Department of Experimental Oncology and Molecular Medicine, Fondazione IRCCS “Istituto Nazionale dei Tumori”, Milan, Italy; ^2^Department of Molecular Virology, Immunology and Medical Genetics, Wexner Medical Center and Comprehensive Cancer Center, The Ohio State University, Columbus, OH, USA; ^3^Department of Anesthesiology, Wexner Medical Center, The Ohio State University, Columbus, OH, USA

**Keywords:** iNKT cell, microRNA, transgenic, thymic development, gene expression regulation

## Abstract

Invariant natural killer T cells (iNKT cells) are CD1d-restricted, lipid antigen-reactive T lymphocytes with immunoregulatory functions. iNKT cell development in the thymus proceeds through subsequent stages, defined by the expression of CD44 and NK1.1, and is dictated by a unique gene expression program, including microRNAs. Here, we investigated whether *miR-155*, a microRNA involved in differentiation of most hematopoietic cells, played any role in iNKT cell development. To this end, we assessed the expression of *miR-155* along iNKT cell maturation in the thymus, and studied the effects of *miR-155* on iNKT cell development using Lck-*miR-155* transgenic mice, which over express *miR-155* in T cell lineage under the lymphocyte-specific protein tyrosine kinase (Lck) promoter. We show that *miR-155* is expressed by newly selected immature wild-type iNKT cells and turned off along iNKT cells differentiation. In transgenic mice, *miR-155* over-expression resulted in a substantial block of iNKT cell maturation at Stage 2, in the thymus toward an overall reduction of peripheral iNKT cells, unlike mainstream T cells. Furthermore, the effects of *miR-155* over-expression on iNKT cell differentiation were cell autonomous. Finally, we identified Ets1 and ITK transcripts as relevant targets of *miR-155* in iNKT cell differentiation. Altogether, these results demonstrate that a tight control of *miR-155* expression is required for the development of iNKT cells.

## Introduction

Invariant natural killer T (iNKT cells) cells are a unique T cell lineage characterized by the expression of a conserved semi-invariant αβ TCR, NK receptors, and by innate effector properties (1). NKT semi-invariant TCR is composed of an invariant α chain (Vα24Jα18 in human beings and Vα14Jα18 in mice), which couples with a limited repertoire of TCR β chains (preferentially Vβ11 in humans and Vβ2, Vβ7, and Vβ8.2 in mouse) (1). The semi-invariant TCR recognizes both self and bacterial glycolipid ligands presented by the antigen-presenting molecule CD1d (2), as well as at least one endogenous peptide involved in multiple disease conditions (3). This wide recognition confers iNKT cells the ability to act as regulators of immune homeostasis (4), as well as sentinels to invading pathogens. Accordingly, iNKT cells play important functions in autoimmune diseases, cancer, infection, and inflammation.

As conventional T lymphocytes, iNKT cells are generated in the thymus from CD4^+^CD8^+^ double positive (DP) precursors, upon stochastic rearrangement of the invariant TCR and positive selection by CD1d-expressing DP thymocytes (5, 6). Positively selected iNKT cells follow a defined developmental program, involving three subsequent stages defined by the expression of CD44 and NK1.1 and the down-regulation of the heat stable antigen (HSA or CD24). In particular, Stage 1 (CD24^lo^CD44^lo^NK1.1^−^) iNKT cells proliferate extensively to expand their pool; Stage 2 (CD24^lo^CD44^hi^NK1.1^−^) iNKT cells become effector-memory cells; Stage 3 (CD24^lo^CD44^hi^NK1.1^+^) iNKT cells up-regulate NK cell receptors such as NK1.1 and become terminally differentiated thymic residents (7, 8). Many factors have been described as crucial for this maturation path, including transcription factors (such as c-Myc, Egr2, Ets1, PLZF, T-bet), signal transducers (such as ITK and Irf1), and other molecules (as WASp and Osteopontin) [reviewed in Ref. (9)]. Emigration of iNKT cells from the thymus occurs mostly at Stage 2 through the lymphotoxin-β and the sphingosine 1-phosphate receptors. The peripheral acquisition of NK markers by iNKT cells is gained via CD1d-dependent mechanisms, but the complete functional maturation requires a final step in which the correct levels of SHP-1 phosphatase in iNKT cells are tuned by CD1d-expressing DCs (10).

MicroRNAs are small endogenous RNAs that play important gene-regulatory roles by pairing to the mRNAs of protein-coding genes to direct their posttranscriptional repression (11). Despite their relatively recent identification, growing evidence indicates that microRNAs are crucial controllers of the programs directing cell differentiation in the immune system, as demonstrated in mice mutants for Dicer, the RNase III enzyme that generates functional microRNAs. In particular, conditional deletion of Dicer causes significant impairment in the generation of functional regulatory T cell subsets, such as FoxP3^+^ regulatory T (Treg) cells (12) and iNKT cells (13). The search for the relevant individual microRNA involved in Treg development and function identified micro-RNA155 (*miR-155*) as a key factor for Treg maintenance. *miR-155* is processed by Dicer from BIC, a non-coding transcript highly expressed in B and T cells and in monocytes/macrophages. In Treg, *miR-155* is directly regulated by FoxP3 and targets suppressor of cytokine signaling 1 (SOCS1), leading to increased sensitivity of IL-2R to IL-2 (14, 15).

On the iNKT cell side, two groups identified *miR-150* as the essential microRNA for thymic and peripheral iNKT cell maturation (16, 17). Notably, Zheng et al. described a partial block in thymic and peripheral iNKT maturation in *miR-150* KO mice, whereas Lanier’s group showed a substantial reduction of iNKT cells in mice over-expressing *miR-150*. These data suggest that a dynamic and tightly regulated expression of *miR-150* is required for optimal iNKT cell development.

Beyond the above-described role in Treg function, *miR-155* has gained attention for its role in cancer. A moderate increase of *miR-155* levels has been observed in many types of malignancies of B cell or myeloid origin, and some of us have shown that transgenic over-expression of *miR-155* in mice results in cancer (18).

Given the relevance of *miR-155* for the homeostasis of the immune system, in this study, we investigated the role of *miR-155* in iNKT cells. Surprisingly, we found that *miR-155* over-expression deeply impacts iNKT cell development, a result that stresses the importance of tight regulation of miRNAs for their correct functioning.

## Materials and Methods

### Mice

C57BL/6 *wild-type* (wt) mice were purchased from Charles River (Italy). Mice were maintained under pathogen-free conditions at the animal facility of Fondazione IRCCS “Istituto Nazionale dei Tumori”. Animal experiments were authorized by the Institute Ethical Committee and performed in accordance to institutional guidelines and national law (DL116/92). Lck-*miR-155* tg mice were generated as previously described (19) and were provided by Dr. Carlo Maria Croce (Wexner Medical Center and Comprehensive Cancer Center, The Ohio State University).

### Cell preparations, antibodies, flow cytometry, and cell sorting

Single-cell suspensions from thymus, liver, spleen, and bone marrow (BM) were prepared as previously described (6).

PerCPCy5.5 anti-HSA (M1/69), APC anti-TCRβ (H57-597), PE-Cy7 anti-NK1.1 (PK136), FITC anti-CD44 (IM7), FITC anti-CD45.1 (A20), PE-Cy7 anti-CD4 (GK1.5), and APC anti-CD8 (53-6.7) were purchased from eBioscience. PBS-57-loaded CD1d-tetramers were kindly provided by NIH Tetramer Core Facility at Emory University (task order # 14724). Surface staining was performed by incubating antibodies and tetramers at 5 μg/ml on ice for 30 min in PBS containing 2% FBS. Flow cytometry data were acquired on a LSR Fortessa (Becton Dickinson) and analyzed with FlowJo software (version 8.8.7; Treestar Inc.).

Invariant natural killer T cells pooled from thymocytes from wt and Lck-*miR-155* tg mice were sorted using a FACSaria (Becton Dickinson) as:

HSA^−^TCRβ^+^tetramer^+^CD44^lo^NK1.1^−^ Stage 1 cells,
HSA^−^TCRβ^+^tetramer^+^CD44^hi^NK1.1^−^ Stage 2 cells,
HSA^−^TCRβ^+^tetramer^+^CD44^hi^NK1.1^+^ Stage 3 cells.

Purity after sorting assessed around 98%.

### Real time RT-PCR

Fifty nanograms of total RNA, isolated by using the miRNeasy miRNA isolation kit (Qiagen), were subjected to reverse transcription according to the manufacturer’s instructions (Applied Biosystems). Quantitative Real time RT-PCR analysis for *miR-155* (assay ID: 002571) was performed according to the TaqMan MicroRNA Assays (Applied Biosystems) and samples normalized by evaluating RNA U6 (assay ID: 001973) expression.

RNA was extracted according to the manufacturer’s instructions (RNeasy MICROKIT, Qiagen) and reverse transcribed using High-Capacity^®^ cDNA Reverse Transcription Kits (Applied Biosystem). Real time RT-PCR were performed on 7900 HT (Applied Biosystem), using TaqMan^®^ Fast Universal PCR masterMix (Applied Biosystem). Assays (Ets1 assay ID: Mm01175819_m1; Itk assay ID: Mm00439862_m1) and samples were normalized by evaluating HPRT1 (assay ID: Mm01545399_m1) expression. Results were obtained using the comparative Ct method.

### BM transplantation

Bone marrow cells were obtained by flushing the cavity of femurs from donor mice. Cells from Lck-*miR-155* tg mice were mixed at 1:1 ratio with CD45.2 wt cells. Lck-*miR-155* recipient mice were lethally γ irradiated with 1000 cGy (given as a split dose 500 + 500 cGy with a 3-h interval). Two hours later, mice were injected i.v. with 10^7^ mixed BM cells. Recipient mice received 0.4 mg/ml gentalyn in the drinking water starting 1 week before irradiation and maintained thereafter.

### Luciferase assay

The 3′-UTRs of human *ITK* and *ETS1* cloned downstream of *Renilla luciferase* gene were purchased from Switchgear Genomics and used according to the manufacturer’s instructions. *Renilla luciferase* assays were performed in MEG-01 cells 48 h after transfection (*n* = 10). The *miR-155* target sites present on *ITK-3*′*-UTR* and *ETS1-3*′*-UTR* target site 1 and target site 2 (underlined) were mutated as indicated below (underlined and bold) using the shown corresponding primers. The mutations were prepared using QuikChange II XL Site-Directed Mutagenesis Kit from Agilent following manufacturer’s protocol. Sequencing of the clones was done to confirm the presence of each mutation. For *ETS1*, the double mutant clone (*ETS-1-M1, 2*) was prepared on the clone were the first *miR-155* target site was previously mutated.

ITK original sequence:

GGATATGTCCTCATTCCATAGAGCATTAGAAGCTGCCACCAGCCCAGG

ITK mutated sequence (M):

GGATATGTCCTCATTCCATAGAGC**GG**TAGAAGCTGCCACCAGCCCAGG

Primers used for mutation:

ITKS 5′-GTCCTCATTCCATAGAGCGGTAGAAGCTGCCACCAG

ITKAS 5′-CTGGTGGCAGCTTCTA**CC**GCTCTATGGAATGAGGAC

ETS original target site 1:

GGACTTAATGTTGAGCTAAGAAGCATTAAGTCTTTGAACTGAATGTATTTTGCATCCC

ETS mutated target site 1 (M1):

GGACTTAATGTTGAGCTAAGAAGC**GG**TAAGTCTTTGAACTGAATGTATTTTGCATCCC

Primers used for mutation:

ETS1S

5′-GGACTTAATGTTGAGCTAAGAAGCGGTAAGTCTTTGAACTGAATG

ETS1AS

5′-CATTCAGTTCAAAGACTTA**CC**GCTTCTTAGCTCAACATTAAGTCC

ETS original target site 2:

GGAGATGAACACTCTGGGTTTTACAGCATTAACCTGCCTAACCTTCATGGTG

ETS mutated target site 2 (M2):

GGAGATGAACACTCTGGGTTTTACAGC**GG**TAACCTGCCTAACCTTCATGGTG

Primers used for mutation of target site 2 (M2):

ETS2S 5′-GAACACTCTGGGTTTTACAGCGGTAACCTGCCTAACC

ETS2AS 5′-GGTTAGGCAGGTTA**CC**GCTGTAAAACCCAGAGTGTTC

The *miR*-Control and *miR-155* were purchased from Life Technologies (catalog number AM17110 and PM12601, respectively). The *miR*-Control molecule is a random sequence, which has been extensively tested in human cell lines and tissues and has no known target transcripts. The *miR*-Control was used as a baseline for evaluating the effects of *miR-155* on Est1 and Itk transcripts.

### Statistical analysis

The graphs and the analysis of data were performed using Prism software (GraphPad Software, Inc.). Results are expressed as means ± SEM or SD. Statistical analysis was performed using a two-tailed Student’s *t*-test with confidence intervals of 95%. Data were considered significantly different at *p* < 0.05 (*p < 0.05, **p < 0.01, ***p < 0.005 by Student’s *t*-test).

## Results

### *miR-155* is down-modulated along thymic iNKT cell maturation

**Figure 1:**
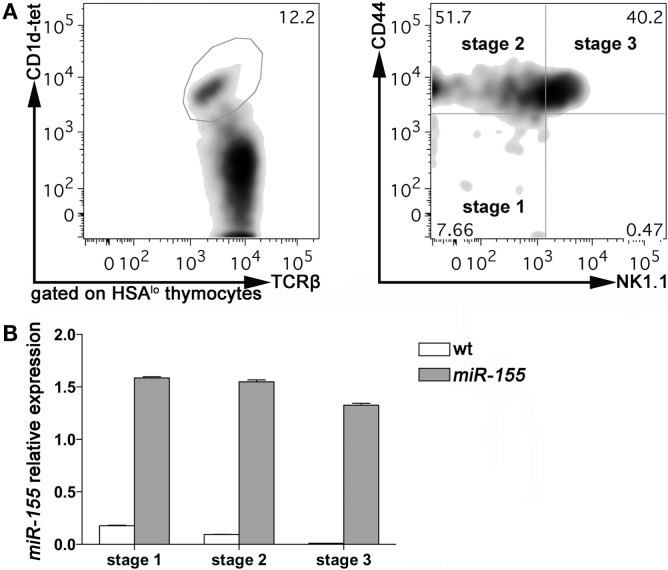
***miR-155* expression in Stage 1, 2, and 3 wt and Lck-*miR-155* tg iNKT cells**. **(A)** Representative plots of the sorting strategy employed to sort wt and *miR-155* tg thymic iNKT cells. iNKT cells were identified as HSA^−^TCRβ^+^CD1d-tetramer^+^ thymocytes, and sorted as Stage 1 (CD44^−^NK1.1^−^), Stage 2 (CD44^+^NK1.1^−^), and Stage 3 (CD44^+^NK1.1^+^) cells. **(B)** Evaluation of *miR-155* levels in sorted cells, as assessed by RT-PCR. White bars represent wt iNKT cells, gray bars represent Lck-*miR-155* tg iNKT cells. The graph shows the pooled results of two independent experiments (in which six mice per group were combined).

To evaluate the expression of *miR-155* in thymic iNKT cells at different stages of maturation, we sorted Stage 1, Stage 2, and Stage 3 iNKT cells from 8 weeks old *wild-type* (wt) C57BL/6 mice, according to PBS-57-loaded CD1d-tetramers, TCRβ, NK1.1, and CD44 staining. Consistently with published literature, Stage 2 and 3 accounted for the majority of iNKT cells at this age, with Stage 1 representing <10% of HSA-negative iNKT cells (Figure 1A). The relative expression of *miR-155* (compared to endogenous control small nuclear RNA U6) was higher in Stage 1, and decreased progressively in Stage 2 and Stage 3 iNKT cells (Figure 1B, white bars). These data indicated that *miR-155* is down-modulated along iNKT cell maturation, suggesting that *miR-155* may be important for early events in iNKT cell lineage instruction, but becomes irrelevant or even detrimental for further maturation. In conventional T cells, *miR-155* is expressed at higher levels by CD4^+^ and CD8^+^ single positive (SP) cells than by CD4^−^CD8^−^ double negative (DN) and CD4^+^CD8^+^ DP thymocytes (Figure S1A in Supplementary Material) and (20), indicating a different regulation exerted on and by *miR-155* in iNKT and T cell subsets.

To assess whether the regulation of *miR-155* along iNKT cell maturation in the thymus is relevant for the development of these cells, we analyzed the effects of a sustained and prominent expression of *miR-155* on iNKT cells development, taking advantage from the Lck-*miR-155* transgenic (tg) mice (19). These mice express *miR-155* at high levels in T lymphocytes beginning at the DN stage (Figure S1B in Supplementary Material), including iNKT cells. Compared to their wt counterparts, Stage 1 iNKT cells from Lck-*miR-155* tg mice expressed sevenfold more *miR-155*, and expression was maintained at the same levels in Stage 2 and 3 (Figure 1B, gray bars). Lck-*miR-155* tg mice thus represent the ideal model to study the significance of *miR-155* down-regulation in iNKT cell development.

### Thymic and peripheral iNKT cell maturation is impaired in Lck-*miR-155* mice

**Figure 2:**
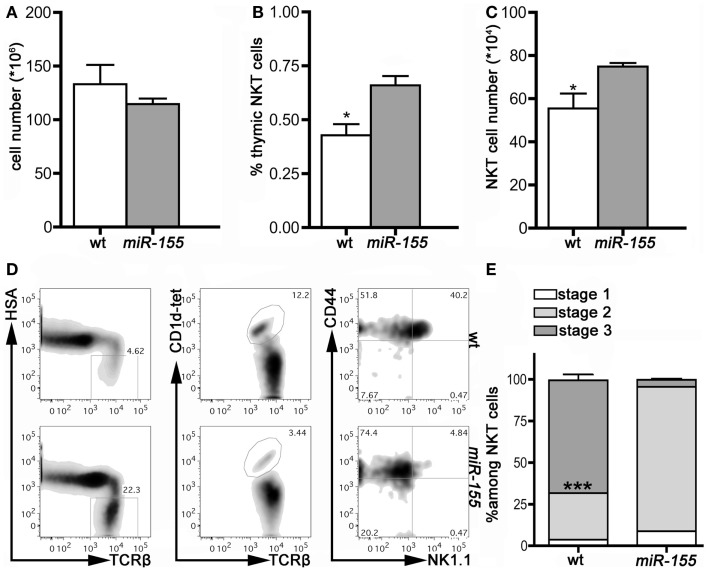
**iNKT cells from Lck-*miR-155* tg thymi display an immature phenotype**. **(A)** Total cell number of thymi isolated from wt (white bar) and Lck-*miR-155* tg mice (gray bar). **(B)** Percentage and **(C)** absolute number of iNKT cells in the thymi of wt (white bar) and Lck-*miR-155* tg mice (gray bar). **(D)** Representative plots of thymic iNKT cells from wt and tg mice. iNKT cells were identified as HSA^−^TCRβ^+^CD1d-tetramer^+^ thymocytes, and then analyzed for CD44 and NK1.1 expression. **(E)** Bars showing the relative distribution in Stage 1, 2, and 3 of wt and *miR-155* tg iNKT cells. White color represents Stage 1, light gray represents Stage 2 and dark gray represents Stage 3 iNKT cells. One representative of three independent experiments with five mice per group. Data are presented as mean ± SEM. *p < 0.05, ***p < 0.005, two-tailed Student’s *t*-test.

We then assessed *miR-155* involvement in iNKT cell development by comparing thymus and peripheral organs of 8 weeks old wt and Lck-*miR-155* tg mice, in terms of iNKT cell frequency, numbers, and phenotype. Interestingly, tg thymi were normal in total cell numbers (Figure 2A), but displayed alterations in the relative distribution of thymocytes in the DN, DP, and SP compartments (not shown), reasonably caused by *miR-155* deregulation in developing tg thymocytes. Thymi from tg mice contained significantly more iNKT cells than wt mice (75 ± 32 × 10^4^ cells in tg versus 55 ± 14 × 10^4^ cells in wt mice, *p* = 0.033) (Figures 2B,C), but the great majority of the tg iNKT pool was constituted by cells with an immature phenotype. In fact, whereas wt iNKT cells were mostly Stage 3 NK1.1^+^ cells, tg iNKT cells encompassed mostly Stage 2 NK1.1^−^ cells (Figures 2D,E), with only few mature NK1.1^+^ cells.

**Figure 3:**
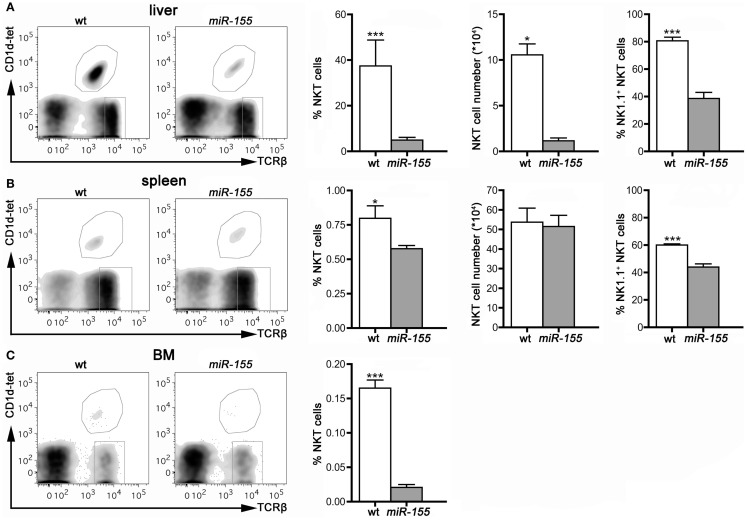
**iNKT cells are reduced in peripheral organs of Lck-*miR-155* tg mice**. Representative stainings and histograms summarizing iNKT cell frequency, absolute number, and NK1.1^+^ expression in liver **(A)**, spleen **(B)** and bone marrow **(C)** of wt and Lck-*miR-155* tg mice. iNKT cells were identified as TCRβ^+^CD1d-tetramer^+^ cells. One representative of three independent experiments with five mice per group. Data are presented as mean ± SEM. *p < 0.05, ***p < 0.005, two-tailed Student’s *t*-test.

In contrast to the higher number of thymic iNKT cells, found in tg mice, peripheral iNKT cells were reduced in all the compartments analyzed, i.e., liver, spleen, and BM (Figures 3A–C), especially if analysis was restricted to mature NK1.1^+^ cells, in comparison to the wt counterparts.

These results indicate that *miR-155* over-expression in T cells arrests iNKT cell maturation at Stage 2 in the thymus and in the peripheral compartments, which results in an overall reduction of iNKT cells in periphery. Therefore, *miR-155* critically regulates iNKT cell differentiation program.

### Defective maturation of thymic NKT cells from Lck-*miR-155* tg mice is cell intrinsic

**Figure 4:**
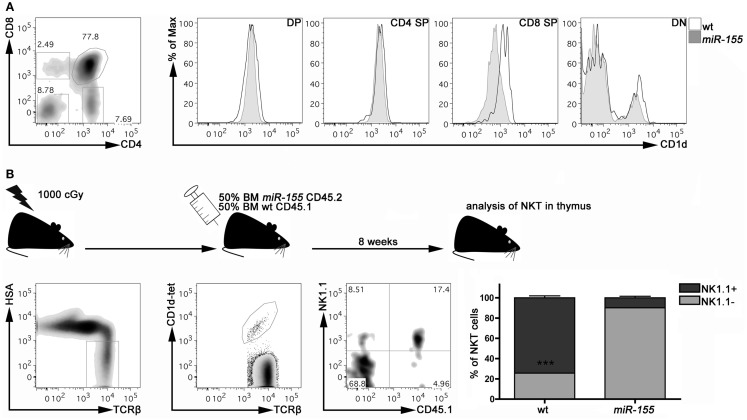
***miR-155* over-expression intrinsically affects iNKT cell maturation in Lck-*miR-155* tg mice**. **(A)** Representative plot of thymic populations according to CD4 and CD8 expression, and CD1d expression on DP, CD4 SP, CD8 SP, and DN cells isolated form the thymus of wt (black line) and *miR-155* tg (gray filled) mice. **(B)** Experimental scheme of mixed BM chimera generation. Eight weeks after transplantation, thymi were collected and frequency and maturation stage of wt (CD45.1) and *miR-155* tg (CD45.2) NKT cells were evaluated. Plots are representative of one mouse of five. Data are presented as mean ± SEM. ***p < 0.005, two-tailed Student’s *t*-test. DP, double positive; CD4 SP, CD4 single positive; CD8 SP, CD8 single positive; DN, double negative; BM, bone marrow.

To dissect the mechanisms by which *miR-155* over-expression impairs iNKT cell differentiation, we first ruled out that *miR-155* over-expression might somehow affect thymic expression of CD1d, the major presenting molecule involved in iNKT cell generation. As shown in Figure 4A, DP, CD4^+^, and DN thymocytes from Lck-*miR-155* tg and wt mice expressed similar levels of CD1d. CD8^+^ thymocytes from Lck-*miR-155* tg mice displayed instead significantly lower levels of CD1d compared to the wt counterpart. As iNKT cells are positively selected by DP thymocytes and no existing data prove a relevant role for CD8^+^ thymocytes in the selection and maturation of iNKT cells, we have reasons to believe that iNKT cell generation is not affected by impaired CD1d expression in CD8^+^ thymocytes in Lck-*miR-155* tg mice.

To determine whether the iNKT cell developmental defect caused by *miR-155* over-expression is instead cell autonomous, we verified whether the development of *miR-155* over-expressing iNKT cells could be rescued by wt thymocytes in mixed BM chimeras. Lethally irradiated Lck-*miR-155* tg mice were reconstituted with an equal mixture of BM cells derived from CD45.1 wt mice and CD45.2 Lck-*miR-155* tg mice. As shown in Figure 4B, in the thymi of the BM chimeras the majority of iNKT cells derived from the CD45.1 wt BM were mature NK1.1^+^ cells. In contrast, iNKT cells derived from the CD45.2 Lck-*miR-155* BM cells mostly displayed an immature NK1.1^-^ phenotype.

Finally, we ruled out that iNKT cells from Lck-*miR-155* tg mice might be characterized by altered homeostasis, impairing their differentiation program in the thymus. As determined by BrdU incorporation *in vivo*, *miR-155* over-expression did not modify the proliferation of thymic iNKT cell in comparison with the wt counterpart (Figure S2 in Supplementary Material).

Thus, impaired iNKT cell maturation caused by *miR-155* over-expression could not be rescued by wt thymocytes; in addition, iNKT cells derived from wt BM developed correctly in the thymic stroma of Lck-*miR-155* tg mice. Collectively, these results indicate a cell-autonomous role for *miR-155* in the control of iNKT cell differentiation.

### Lck-*miR-155* tg iNKT cells fail to up-regulate Ets1 and Itk upon maturation

The search for the potential *miR-155* targets in iNKT cells identified Ets1 and Itk (inducible T cell kinase) molecules as the most likely candidates. Ets1 is a member of the Ets winged helix-turn-helix transcription factor family. Itk belongs to the Tec family of non-receptor tyrosine kinases, which plays a significant role in signaling downstream of the TCR. Although in different cell types, both Ets1 (21) and Itk (22) have been shown to represent direct targets of *miR-155* regulation. Both Ets1 KO (23, 24) and Itk KO (25) mice display a severe impairment in iNKT cells, characterized by an arrest at Stage 2 that closely resembles the condition of Lck-*miR-155* tg mice. Considering the relevance of both Ets1 and Itk in iNKT differentiation and the regulatory roles exerted by *miR-155* on these two genes, we investigated them further.

**Figure 5:**
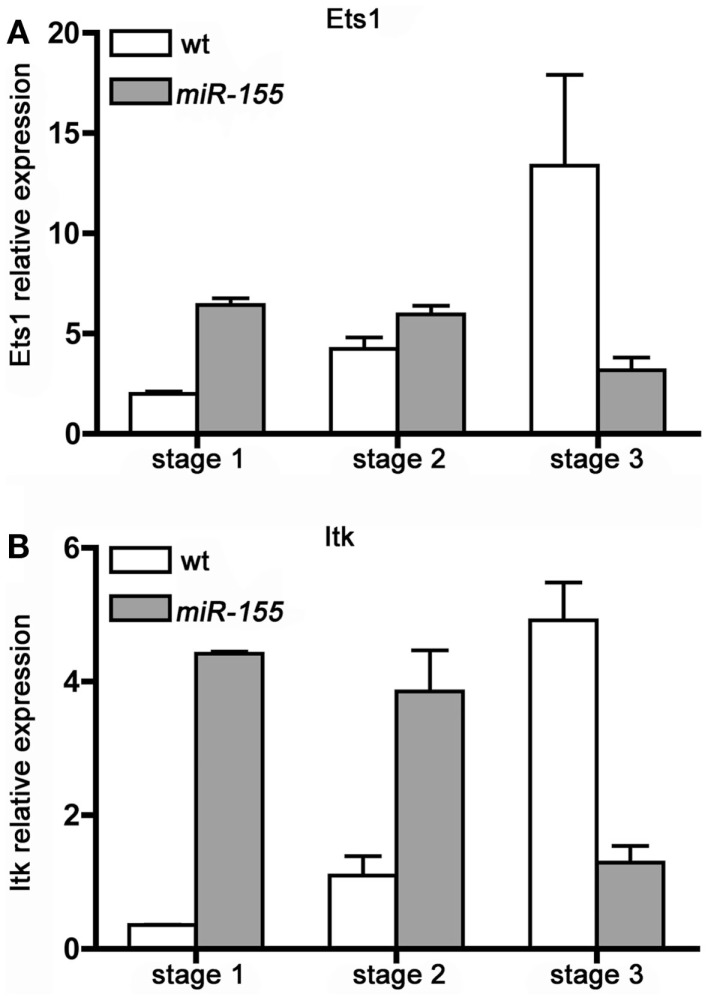
**iNKT cells over-expressing *miR-155* fail to up-regulate Ets1 and Itk upon maturation**. **(A)** Ets1 and **(B)** Itk transcripts expression level was evaluated in sorted Stage 1, 2, and 3 iNKT cells isolated from wt (white bars) and Lck-*miR-155* tg (gray bars) mice. Along wt iNKT development both Ets1 and Itk are up modulated, mirroring the down-modulation of *miR-155*. On the contrary in *miR-155* tg iNKT, the expression of both transcripts is impaired. Data are representative of two independent experiments, each counting five mice for group.

We determined the expression of Ets1 and Itk transcripts in Stage 1, 2, and 3, iNKT cells isolated from wt and Lck-*miR-155* tg mice. As shown in Figure 5, wt iNKT cells up-regulate both transcripts upon maturation from Stage 1 to 2 and 3: in particular, Ets1 increases up to 13-fold from Stage 1 to Stage 3, whereas Itk has a 5-fold induction. In contrast, in iNKT cells from Lck-*miR-155* tg mice, Stage 1 cells have a four to sixfold higher expression of both transcripts compared to the wt counterparts, but their expression decreases upon maturation, resulting in a severe reduction of Ets1 and Itk expression in Stage 3 cell compared to wt cells. The defective down-modulation of Ets1 and Itk at the Stage 1 and 2 of *miR-155* tg iNKT cells might be due to the presence of a shorter isoform of their 3′UTR that may occur during differentiation/maturation (26).

In the wt setting, Ets1 and Itk up-regulation along iNKT cell thymic maturation is paralleled by a concomitant decrease in *miR-155* levels (Figure 1B); in tg iNKT cells, which over-express *miR-155* (Figure 1B), expression of Ets1 and Itk is instead consistently down-modulated at least in Stage 3. These results strongly suggest the existence of a regulatory function exerted by *miR-155* over Ets1 and Itk in iNKT cell maturation.

### Ets1 and Itk are direct *miR-155* targets in iNKT cells

**Figure 6:**
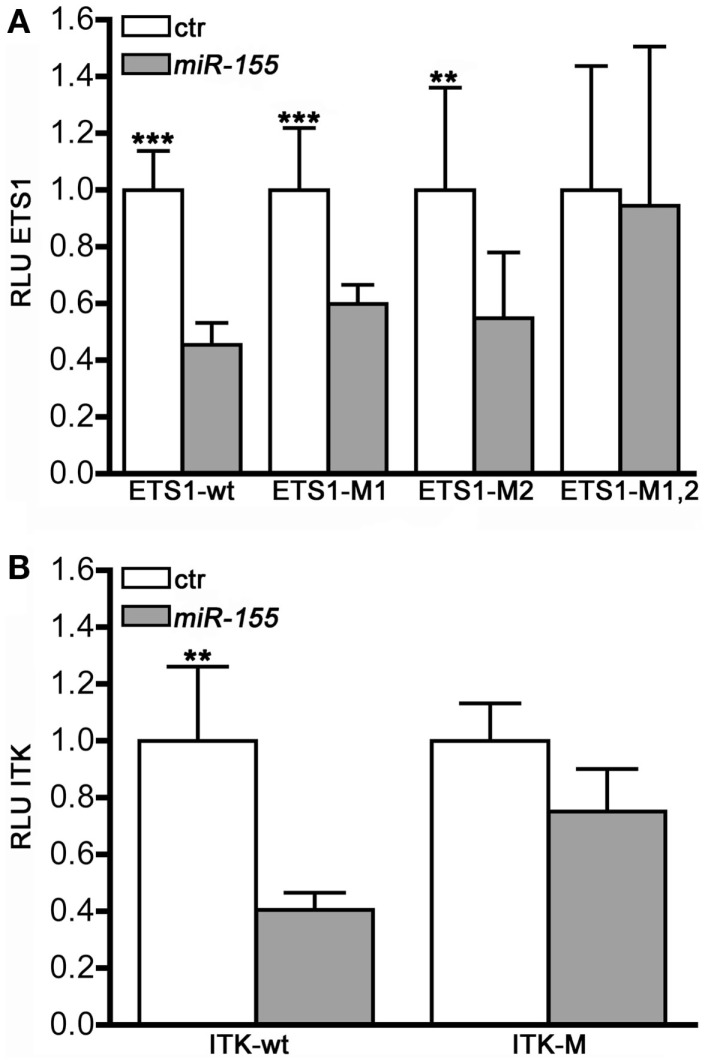
**Ets1 and Itk are direct targets of *miR-155***. MEG-01 cells were co-transfected with luciferase reporter constructs containing either the wild-type or the mutated **(A)** ETS1-, and **(B)** ITK1-3′UTRs and with either *miR-155* or *miR*-Control (ctr) and assessed for luciferase activity (RLU) 48 h after the transfection (*n* = 10).

Function and immune response activities of *miR-155* are conserved in both mouse and human (27). To further validate that *miR-155* modulates iNKT cell maturation through the targeting of Ets1 and Itk transcripts, the 3′UTRs of Ets1 and Itk were cloned downstream of *Renilla luciferase* gene and the effects of *miR-155* were assayed on luciferase reporter assays. Cotransfection of the wt constructs, or either Luciferase-Ets1-3′UTR or Luciferase-Itk-3′UTR, with *miR-155* resulted in the reduction of the luciferase activity compared to the effects of *miR*-control. *miR-155* over-expression also significantly reduced the expression of Luciferase-Ets1-3′UTR-M1 and Luciferase-Ets1-3′UTR-M2 constructs, each containing a single mutated *miR-155* site, suggesting that each of the *miR-155* target site in the Ets1 clone is functional. These effects were abolished when both putative *miR-155* target seed sites were mutated on the Luciferase-Ets1-3′UTR (Figure 6A). Mutation of *miR-155* target site on the Itk transcript also impaired the *miR-155* downregulating effects on the Luciferase-Itk-3′UTR expression (Figure 6B). Altogether, these data demonstrate that *miR-155* directly targets Ets1 and Itk transcripts, and further establish *miR-155* as a key regulator of iNTK differentiation.

## Discussion

Invariant natural killer T cells are unconventional T cells, and, as such, follow a unique differentiation program. Upon positive selection by CD1d expressed on immature DP thymocytes, iNKT maturtion passes through three developmental stages according to CD44 and NK1.1 expression. This maturation process is strictly controlled by transcription factors, signals transduced (9), and microRNA (13).

Our study reveals for the first time a novel mechanism of control in iNKT cell maturation process, which involves the physiological decrease of *miR-155*, such to ensure the up-regulation of its targets (Itk and Ets1), and therefore proper iNKT lymphocyte maturation. We show that abundant and sustained expression of *miR-155* in immature and mature T cells results in a dramatic defect in late-stage maturation of iNKT cells, and accordingly reduced number of iNKT cells in the peripheral compartments. Our data integrate previous studies on the control of iNKT cell physiology by microRNAs, and indicate that a complex and coordinated interaction with different microRNAs and their target is likely involved in the pathway of iNKT cell differentiation.

The crucial role exerted by *miR-155* in regulating lymphocyte biology was demonstrated in a B cell restricted *miR-155* transgenic mouse model. In these mice, the over-expression of *miR-155* under the Eμ promoter caused uncontrolled pre-B cell proliferation followed by high-grade lymphoma/leukemia (18).

Interestingly, despite the dramatic consequences of *miR-155* over-expression in B cells, none of the Lck-*miR-155* tg mice under our observation developed thymomas or peripheral malignancies, suggesting that *miR-155* regulates different targets in B and T lymphocytes, and that the targets in T cells might not necessary be tumor suppressor genes. It is also highly probable that the levels of *miR-155* transgene expression under the Lck promoter did not reach the high levels reached by Eμ promoter in B cells. As the effects of the microRNAs are often dose dependent this might explain the lack of leukemogenesis in Lck-*miR-155* tg mice.

Although the over-expression of *miR-155* in Lck-*miR-155* mice does not cause a neoplastic transformation, several studies highlighted the importance of a regulated expression of this microRNA in both CD4^+^ and CD8^+^ T lymphocytes (28). In particular, it was demonstrated that in CD4^+^Foxp3^+^ regulatory T cells, *miR-155* regulate SOCS1, intervening in the loop that, starting from FoxP3 and through SOCS1, leads to a sustained IL-2R signaling, necessary for Treg homeostasis (14).

In light of these observations, we extended the study of *miR-155* to iNKT lymphocytes. We identified two targets of *miR-155* in iNKT cells: Ets1 and Itk. Several studies have contributed to demonstrate that numerous molecules downstream the TCR are key for the development of iNKT cells, which strongly relies on the strength of TCR signaling in response to cognate interaction with CD1d. ITK is a member of the Tec family of non-receptor protein tyrosine kinases, which includes Rlk and Tec, and is important for effective signaling through the TCR. In the absence of ITK, iNKT cells are reduced in the thymus and periphery, and in both compartments they show a defective NK1.1 up-regulation, indicative of a failure to progress to Stage 3 (29). Defective ITK expression has been linked to an impaired induction of the transcription factor T-bet, the master regulator of iNKT cell maturation (30). Our data from Lck-*miR-155* tg mice phenocopy those obtained in Itk KO mice, and show opposite expression levels of *miR-155* and ITK in developing iNKT cells. Moreover, in both Itk KO and Lck-*miR-155* tg mice, CD8 SP cells display reduced CD1d expression; this finding has probably no functional meaning, but constitutes an additional indication of the actual interaction between *miR-155* and Itk in thymocytes. Similarly, Ets1 was also linked to the T cell maturation, via controlling the expression of TCRα gene (31).

We propose that *miR-155* acts in itself as a modulator of TCR strength signaling by modulating the levels of Itk and Ets1 and consequently modulating iNTK cell maturation.

In conclusion, our study supports a novel regulatory role for *miR-155* in the unique developmental program of iNKT cells and suggests that a dynamic regulation of *miR-155* levels is critical for the physiology of these immunoregulatory cells.

## Author Contributions

AB, PP, CC, and MC designed the study, AB, PP, ET, and AR performed research, SC generated the Lck-*miR-155* tg mice. AB, PP, and ET wrote the paper.

## Conflict of Interest Statement

The authors declare that the research was conducted in the absence of any commercial or financial relationships that could be construed as a potential conflict of interest.

## Supplementary Material

The Supplementary Material for this article can be found online at http://journal.frontiersin.org/article/10.3389/fimmu.2015.00140
