# Supplementary material for: Regulated Expression of miR-155 is Required for iNKT Cell Development
Source: Front Immunol. 2015 Mar 30;6:140. doi: 10.3389/fimmu.2015.00140 (PMC4378312; doi:10.3389/fimmu.2015.00140)
Supplement: Supplementary file 1 [file Image_1.PDF]

Supplementary Material

Regulated expression of *miR-155* is required for iNKT cell development

Alessia Burocchi[1+], Paola Pittoni[1+], Esmerina Tili[2,3], Alice Rigoni[1], Stefan Costinean[2], Carlo M. Croce[2] and Mario P. Colombo[1*]

[1]Molecular Immunology Unit, Department of Experimental Oncology and Molecular Medicine, Fondazione IRCCS "Istituto Nazionale dei Tumori", Via Amadeo 42, 20133 Milan, Italy;
[2] Department of Molecular Virology, Immunology and Medical Genetics, The Ohio State University Wexner Medical Center and Comprehensive Cancer Center, 460 W. 12th Ave., Columbus, OH 43210, USA;
[3]Department of Anesthesiology, Wexner Medical Center, The Ohio State University;

+ Equally contributing authors

*Correspondence:
Mario P. Colombo
Fondazione IRCCS "Istituto Nazionale Tumori"
Department of Experimental Medicine
Molecular Immunology Unit
Via Amadeo 42
I-20133 Milan, Italy.
mario.colombo@istitutotumori.mi.it

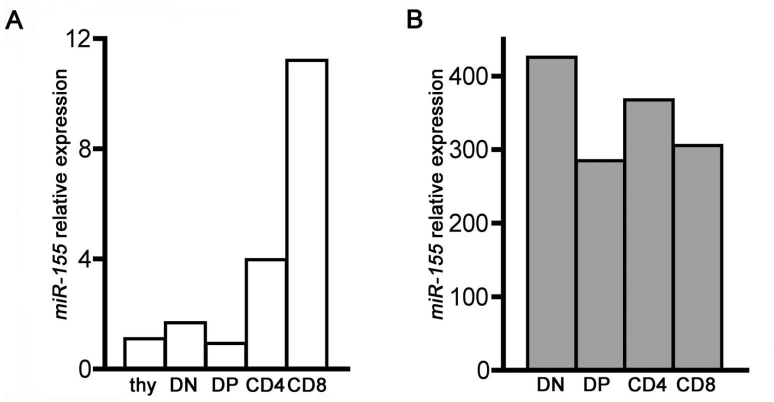

Fig. S1

Figure S1: modulation of *miR-155* expression in thymus. *miR-155* expression in T cell subsets from **(A)** wt and **(B)** *miR-155* tg thymi by RT-PCR. Data are from 2 independent experiments, pooling 3 mice per group. DN: double negative, DP: double positive, SP: single positive, thy: total thymus wt.

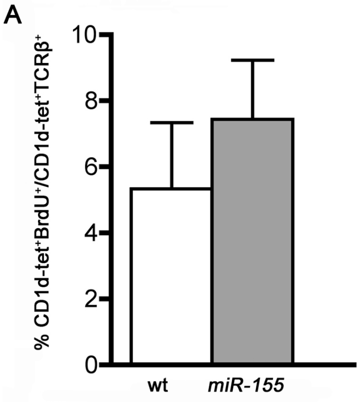

Figure S2: proliferation of thymic iNKT cells in wt and Lck-*miR-155* tg mice.

Frequency of proliferating wt and *miR-155* tg BrdU-positive TCRβ$^+$CD1d-tet$^+$ iNKT cells. One representative of 2 independent experiments with 4 mice per group is shown. Data are presented as mean ± SEM.
